# The change rate in serum nitric oxide may affect lenvatinib therapy in hepatocellular carcinoma

**DOI:** 10.1186/s12885-022-10002-x

**Published:** 2022-08-23

**Authors:** Atsushi Kawamura, Haruki Uojima, Makoto Chuma, Xue Shao, Hisashi Hidaka, Takahide Nakazawa, Akira Take, Yoshihiko Sakaguchi, Kazushi Numata, Makoto Kako, Akito Nozaki, Shintaro Azuma, Kazue Horio, Chika Kusano, Koichiro Atsuda

**Affiliations:** 1 Department of Pharmacy, Kitasato University Hospital, Sagamihara, Kanagawa Japan; 2 Department of Gastroenterology, Internal Medicine, Kitasato University School of Medicine, 1-15-1 Kitasato, Minami-ku, Sagamihara, Kanagawa 252-0375 Japan; 3 Gastroenterology Medicine Center, Shonan Kamakura General Hospital, Kamakura, Kanagawa Japan; 4 Department of Gastroenterology, Yokohama City University Hospital, Yokohama, Kanagawa Japan; 5 Gastroenterological Center, Yokohama City University Medical Center, Yokohama, Kanagawa Japan; 6 Nakazawa Internal Medicine Clinic, Sagamihara, Kanagawa Japan; 7 Department of Microbiology, Kitasato University School of Medicine, Sagamihara, Kanagawa Japan; 8 School of Pharmaceutical Sciences, Kitasato University, Shirokane, Minato-ku, Tokyo Japan

**Keywords:** Nitric oxide, Lenvatinib therapy, Hepatocellular carcinoma, Adverse effects, Progression-free survival, Overall survival

## Abstract

**Background:**

Lenvatinib is appropriate for reducing the production of nitric oxide (NO) and facilitating as block angiogenesis. However, to our knowledge, there are no data that support the correlation between NO and clinical response in patients who received lenvatinib therapy for HCC. Therefore, we investigated the correlation between the change rate of NO levels and clinical responses including adverse events (AEs) after lenvatinib therapy for unresectable hepatocellular carcinoma (HCC).

**Methods:**

This study was conducted using previously collected data from another study. We enrolled 70 patients who received lenvatinib for advanced or unresectable HCC. NO was measured by converting nitrate (NO_3_^−^) to nitrite (NO_2_^−^) with nitrate reductase, followed by quantitation of NO_2_^−^ based on Griess reagent. To determine whether lenvatinib influences NO in unresectable HCC, we evaluated the influence of the change rate of NO from baseline after administration of lenvatinib on maximal therapeutic response and SAE.

**Results:**

After lenvatinib administration, a change rate in the NO from 0.27 to 4.16 was observed. There was no difference between the clinical response to lenvatinib and the change rate of NO (*p* = 0.632). However, the change rate of NO was significantly lower in patients with AEs than in those without AEs (*p* = 0.030). When a reduction in NO rate of < 0.8 was defined as a clinically significant reduction of NO (CSRN), the CSRN group had significantly worse progression-free survival (PFS) and overall survival (OS) than the non-CSRN group (*p* = 0.029 and *p* = 0.005, respectively).

**Conclusion:**

Decreased NO levels were associated with the occurrence of AEs and worse prognosis after lenvatinib administration. Change rate in serum NO can be used as predictive markers in patients receiving lenvatinib therapy for HCC.

**Supplementary Information:**

The online version contains supplementary material available at 10.1186/s12885-022-10002-x.

## Introduction

Malignant tumors require the formation of mature blood vessels to promote their growth and contribute to pathological processes in the tumor environment [1, 2]. Nitric oxide (NO), a simple gas with divergent biological activities, seems to play a crucial role in angiogenesis [3]. The tumor-promoting effect of NO is understood as a convergence of diverse signaling mechanisms with prominent pathways such as NO synthase (NOS)-derived NO and vascular endothelial growth factor (VEGF) [4, 5].

VEGF is secreted by tumor cells in response to hypoxia. Exposure of endothelial cells to VEGF leads to the phosphorylation and activation of NOS, resulting in the conversion of L-arginine and molecular oxygen into L-citrulline and NO [6]. Thus, VEGF elevation has been extensively reported to correlate with angiogenesis and tumor progression [7]. VEGF expression correlates with the degree of tumor vascularization and increased metastatic risk [5].

On this basis, simultaneously suppressing VEGF signals suppress tumor angiogenesis to the cancer cells, and VEGF inhibitors appropriately reduce the production of NO and facilitate antitumor drug delivery as block angiogenesis [8, 9]. Lenvatinib, a novel multikinase inhibitor that targets VEGF receptors, reduces NO production by reducing the activity of angiogenic factor-mediated pathways [10, 11]. A randomized phase III non-inferiority trial showed lenvatinib was non-inferior to sorafenib in overall survival (OS) for the patients with unresectable hepatocellular carcinoma (HCC) [12].

Assuming more dramatic effects in unresectable HCC, the response to this drug is unpredictable. However, a previous study suggested that elevated NO levels in HCC patients were significantly reduced after radiofrequency ablation [13]. Lenvatinib reduce NO production by reducing the activity of NOS-derived NO and VEGF. Therefore, we hypothesized that there was a positive correlation between the reduction of the NO levels and the therapeutic effect of lenvatinib for HCC. Furthermore, NO is a vital molecule that contributes to numerous physiological phenomena in various biological systems [14]. Therefore, severe reduction of NO levels can lead to adverse events (AEs) from simultaneous suppression of vital organs after lenvatinib therapy. However, to our knowledge, there are no data that support the correlation between NO and a clinical response in patients who received lenvatinib therapy for HCC. Therefore, we investigated the correlation between the change rate of the NO levels and the clinical responses including adverse events (AEs) after lenvatinib therapy for unresectable hepatocellular carcinoma (HCC).

## Methods

### Ethics

This study was approved by the Institutional Review Boards and Ethics Committees of all hospitals involved (IRB number: 11000845). The study was registered in the Japan Registry of Clinical Trials (jRCT ID: 1030210283). These data were previously collected under another study (UMIN ID: 000036625). The data were collected after each patient wrote informed consent for the treatment.

### Patients

**Fig. 1:**
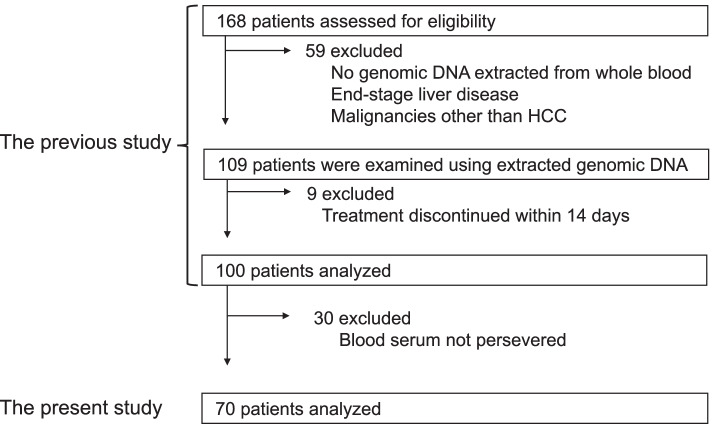
Study flow diagram

This study was performed using previously collected data under another study that was conducted across three medical institutions in Japan from 2017 to 2020 [15]. The preliminary study enrolled 168 patients aged > 20 years who received lenvatinib for advanced or unresectable HCC (Fig. 1). Of those, 68 patients were excluded due to the following exclusion criteria: (i) lenvatinib discontinued within 14 days, (ii) malignancies other than HCC, (iii) no genomic DNA extracted from blood, and (iv) end-stage liver failure. Therefore, the previous study analyzed 100 patients [15]. Of those patients, 30 did not receive blood serum to identify the biomarkers of the response of HCC to lenvatinib. Therefore, relevant clinical data were collected from the remaining 70 patients.

### Diagnosis of HCC and recommendation of lenvatinib

HCC diagnosis was based on imaging modalities such as computed tomography and magnetic resonance imagery. Liver tumors with atypical imaging findings were histopathologically analyzed by biopsy. Lenvatinib was administered for unresectable or advanced HCC that was characterized by vascular invasion, metastatic disease, and/or progression after locoregional treatments.

A starting drug dose was dependent on the patient’s weight: 12 mg and 8 mg/day for body weight ≥ 60 kg and <  60 kg, respectively. However, for patients with risk factors, such as low PS (performance status), Child-Pugh class B, and sarcopenia, an approved dose reduction from the initial dose depended on the attending physician’s discretion.

### NO measurement

Blood samples, collected at a pretreatment visit and within a month after administration of Lenvatinib [16], were centrifuged at 25 °C (room temperature) at 3000 rpm for 5 min. The fractionated serum was stored at − 45 °C. NO was measured by converting nitrate (NO_3_^−^) to nitrite (NO_2_^−^) with nitrate reductase, followed by quantitation of NO_2_^−^, using a Griess reagent. In this experiment, we used a colorimetric Nitric Oxide Assay kit (Oxford Biomedical Research, MI, USA). Absorbance was measured at 540 nm using a microplate reader (see Additional file 1). We measured the change levels and rate in NO from baseline to after administration of lenvatinib. The change rate in NO was calculated as in the NO levels after lenvatinib therapy and compared with the previous NO levels.

### End-point measurement

The end-points were the influence to the maximal therapeutic response and Serious AE (SAE) from the change levels and rate in NO after administration of lenvatinib. The maximal therapeutic response was evaluated by the modified Response Evaluation Criteria in Solid Tumors (mRECIST). SAEs were defined as events that result in death, are life threatening, require inpatient hospitalization or prolongation of existing hospitalization, or result in persistent or significant disability/incapacity according to ICH (International Council for Harmonisation of Technical Requirements for Pharmaceuticals for Human Use).

### Statistical analyses

All *p*-values were two-sided, with differences < 0.05 considered to indicate statistical significance. Two categorical variables in the population were examined using the Chi-square test. The differences in OS and progression-free survival (PFS) were evaluated with the log-rank test and the Kaplan-Meier method in the two groups. PFS and OS were analyzed by the Cox proportional hazards regression model. The correlation between NO levels and the response to lenvatinib was analyzed by calculating the odds ratio and 95% confidence interval (CI) using univariate and multivariate logistic regression analyses. All analyses were performed using SPSS (version 24.0; IBM Corporation, Armonk, NY, USA). Statistical analyses were reviewed by the Statista Corporation, Kyoto, Japan.

## Results

### Patients’ characteristics

Table 1 shows the patients’ baseline characteristics. The mean age was 71.5 ± 8.9 years, and 53 of 70 patients (75.7%) were male. The mean body weight was 61.5 ± 12.2 kg. The causes of chronic liver disease were virus (*n* = 38), nonalcoholic fatty liver disease (*n* = 16), alcohol (*n* = 10), and others (*n* = 6). There were 46 patients (65.7%) with liver cirrhosis. Lenvatinib was administered for vascular invasion (*n* = 24), metastatic disease (*n* = 22), and progression after locoregional treatment (*n* = 46). The types of treatment given were radiofrequency ablation (RFA) (*n* = 28), transcatheter arterial therapy (*n* = 50), and molecular targeted therapy (*n* = 6). There were 32 (45.7%) and 38 (54.3%) patients who were diagnosed as having Barcelona Clinic Liver Cancer (BCLC) stages B and C, respectively. There were 25, 35, and 10 patients who had daily initial doses of 12 mg, 8 mg, and 4 mg, respectively. The 10 patients, who received an initial dose of 4 mg, had liver cirrhosis with sarcopenia.

**Table 1:** Baseline clinical characteristics

	N	70
Age	**yrs**	**71.5 ± 8.9**
Gender: Male	**n (%)**	**53 (75.7)**
Etiology: HBV/HCV/Alcohol/NASH/etc.	**n**	**10/28/10/16/6**
Performance status: 0/1	**n**	**65/5**
Child-Pugh score 5:6:7:8:9	**n**	**39:17:9:4:1**
Child-Pugh class: A/B	**n**	**56/14**
Weight	**kg**	**61.5 ± 12.2**
< 60 kg/≥ 60 kg		**37/33**
Body mass index	**kg/m**^**2**^	**23.3 ± 4.6**
Barcelona Clinic Liver Cancer stage: B/C	**n**	**32/38**
Macroscopic portal vein invasion: Yes/No	**n**	**24/46**
Extrahepatic spread: Yes/No	**n**	**22/48**
Up to 7: In/Out	**n**	**36/34**
Tumor size:	**mm**	**47.1 ± 38.9**
Previous therapy:		
Radiofrequency ablation	**n**	**28**
Transcatheter treatment	**n**	**50**
Molecularly-targeted therapy	**n**	**6**
Initial dose of lenvatinib: 4 mg/8 mg/12 mg	**n**	**10/35/25**
Hemoglobin	**g/dL**	**12.2 ± 2.12**
Platelets	**× 10**^**4**^**/μl**	**14.1 ± 6.3**
Prothrombin time	**%**	**87.1 ± 19.3**
Serum albumin	**g/dL**	**3.7 ± 0.45**
BUN	**g/dL**	**20.1 ± 14.3**
Serum creatinine	**mg/dL**	**0.86 ± 0.36**
Aspartate aminotransferase	**IU/L**	**52.0 ± 30.3**
Alanine aminotransferase	**IU/L**	**34.0 ± 23.8**
Total bilirubin	**g/dL**	**0.9 ± 0.41**
Ammonia	**μg/dl**	**51.2 ± 43.0**
α-fetoprotein	**ng/mL**	**12,309 ± 49,567**
PIVKA-II	**mAU/mL**	**6083 ± 14,136**

### Clinical responses of patients who received lenvatinib

The numbers of complete response, partial response, stable disease, progressive disease (PD), and unevaluated response in maximal therapeutic response were 2 (2.9%), 24 (34.2%), 25 (35.7%), 15 (28.0%), and 4 (5.7%), respectively. The median PFS and OS in all patients were 163 and 403 days (95% CI 124–238 and 311–524 days), respectively. The median time to treatment failure values for all patients was 247 days (95% CI: 201–292). Reasons to discontinue treatment were PD (*n* = 40), unmanageable AEs (*n* = 26), and withdrawal at the patients’ own discretion (*n* = 4). Table 2 shows the treatment-related severe AEs in the study period. After discontinuation of lenvatinib therapy, administration of another molecularly targeted therapy, transcatheter treatment, and best supportive care were performed for 18 (25.7%), 8 (11.4%), and 29 (41.4%) patients, respectively.

**Table 2:** Severe adverse events

Severe AE	CSRN: *n* = 25	Non-CSRN: *n* = 45
Decreased appetite	**6 (24.0)**	**4 (8.8)**
Hepatic ascites	**4 (16.0)**	**3 (6.6)**
Hepatic encephalopathy	**3 (12.0)**	**2 (4.4)**
Gastrointestinal bleeding	**2 (8.0)**	**–**
Proteinuria	**2 (8.0)**	**1 (2.2)**
Increased blood bilirubin	**1 (4.0)**	**1 (2.2)**
Acute pancreatitis	**1 (4.0)**	**1 (2.2)**
Rhabdomyolysis	**1 (4.0)**	**–**
Sepsis	**1 (4.0)**	**–**
Gastrointestinal perforation	**–**	**1 (2.2)**
Interstitial pneumonia	**–**	**1 (2.2)**

### Pretreatment NO levels

The mean NO level at baseline was 49.2 ± 39.8 nmol/mL. We analyzed the correlation between the NO levels and baseline characteristics (see Additional file 2). BCLC stages B and C were 48.3 ± 40.5 and 50.0 ± 39.8, respectively. Up to 7 in and out were 46.6 ± 39.8 and 52.0 ± 40.6, respectively. The NO levels in patients with and without high blood pressure (HBP) were 49.3 ± 41.2 and 49.1 ± 39.3, respectively. No significant differences were found among age, sex, body weight, etiology, tumor size, HBP, BCLC stage, and presence of portal invasion.

### Change levels and rate of NO after lenvatinib therapy

**Fig. 2:**
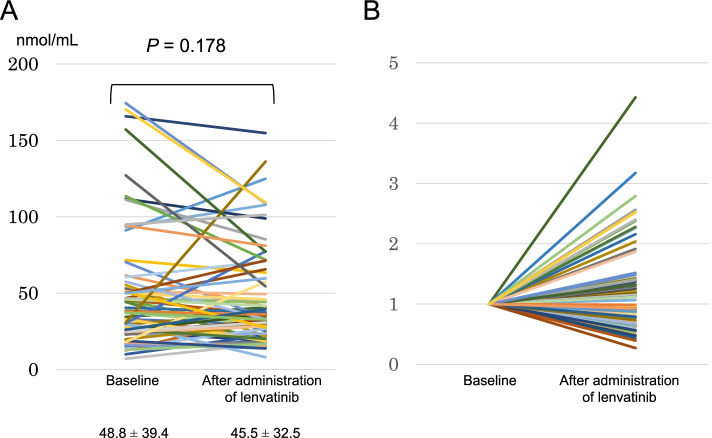
**A** Change levels of NO after lenvatinib therapy “pre” indicates before lenvatinib administration; “post” indicates after lenvatinib administration. **B** Change rate of NO after lenvatinib therapy “pre” indicates before lenvatinib administration; “post” indicates after lenvatinib administration

Lenvatinib reduced the NO levels in 39 (55.7%) patients. After lenvatinib administration, change levels in the NO from 49.2 ± 39.8 to 45.1 ± 32.5 nmol/mL were observed (*p* = 0.193) (Fig. 2A) and a change rate in the NO was observed from 0.27 to 4.16 (Fig. 2B).

#### Tumor response in the change levels and rate of NO after administration of lenvatinib

**Fig. 3:**
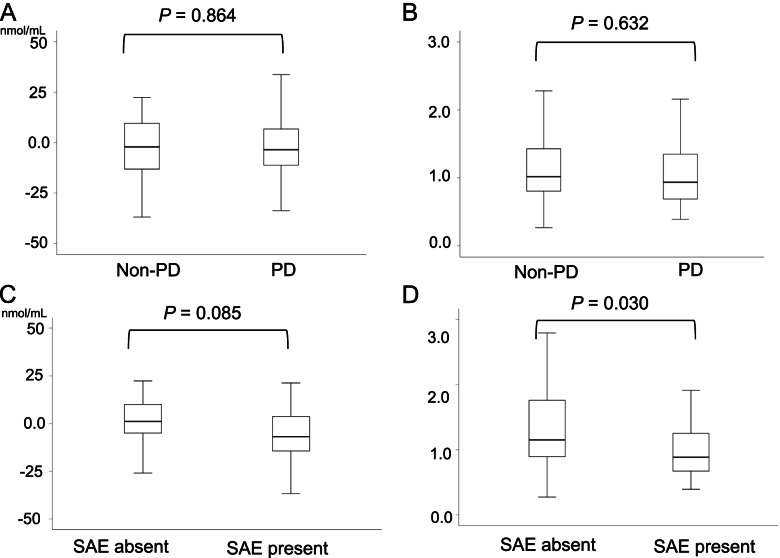
**A** Comparisons between PD and non-PD in the change levels of NO after administration of lenvatinib. **B** Comparisons between PD and non-PD in the change rate of NO after administration of lenvatinib. **C** Comparisons between the absence and presence of SAE in the change levels of NO after administration of lenvatinib. **D** Comparisons between the absence and presence of SAE in the change rate of NO after administration of lenvatinib

Non-PD and PD were − 4.4 ± 28.5 and − 3.2 ± 19.9 in the change levels of the NO. There was no difference between clinical response to lenvatinib therapy and change levels of the NO (*p* = 0.864) (Fig. 3A). Non-PD and PD were 1.3 ± 0.8 and 1.2 ± 0.7, in the change rate of the NO. There was no significant difference between clinical response to lenvatinib therapy in HCC and change rate of the NO (*p* = 0.632) (Fig. 3B).

#### SAE in the change levels and rate of NO after administration of lenvatinib

The change levels of the NO in the patients with the absence and presence of SAE were 1.12 ± 30.6 and − 9.67 ± 19.6. The change levels of NO was lower in patients with AEs than in those without SAE (*p* = 0.085) (Fig. 3C). The change rate of the NO in the patients with the absence and presence of SAE were 1.4 ± 0.9 and 1.0 ± 0.52, respectively. The change rate of NO was significantly lower in patients with SAE than in those without SAE (*p* = 0.030) (Fig. 3D).

### Correlation between HBP and change of NO after lenvatinib therapy

The change levels of NO in patients with and without HBP were − 4.1 ± 18.1 and − 3.9 ± 21.9, respectively. No significant correlation was found between the presence of HBP and change levels of NO (*p* = 0.561). Furthermore, the change rate of NO in patients with and without HBP were 1.1 ± 1.0 and 1.2 ± 0.9, respectively. No significant correlation was found between the presence of HBP and change rate of NO (*p* = 0.813).

### PFS and OS based on reduced NOS levels

Receiver-operating characteristic (ROC) curve analysis was performed to assess the occurrence of SAE in patients with HCC. The respective cut-off points for SAE after lenvatinib treatment were estimated using ROC curves for the change rate of the NO (see Additional file 3). Using a cut-off for the reduction of 0.8, predicting the occurrence of AEs had a sensitivity of 77.3% and a specificity of 42.3%. A reduction in NO rate of < 0.8 was defined as a clinically significant reduction of NO (CSRN).

**Fig. 4:**
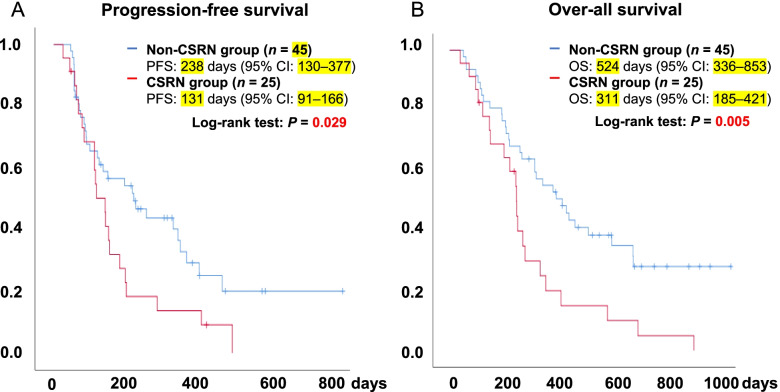
PFS and OS in the patients with HCC based on the CSRN. **A** The median progression free survival in the CSRN and non-CSRN groups in patients with HCC. **B** The median overall survival in the CSRN and non-CSRN groups in patients with HCC

The median PFS in the CSRN and non-CSRN groups was 131 days and 238 days (95% CI 91–166 days and 130–377 days), respectively. Patients in the CSRN group experienced significantly worse PFS than those in the non-CSRN group (log-rank test for trend: PFS, *p* = 0.029) (Fig. 4A).

The median OS in the CSRN and non-CSRN groups was 311 and 524 days (95% CI 185–421 days and 336–853 days), respectively. Patients in the CSRN group experienced significantly worse OS than those with non-CSRN (log-rank test for trend: OS, *p* = 0.005) (Fig. 4B).

### Univariate and multivariate analysis of factors affecting PFS and OS

Table 3 shows the risk factors associated with PFS using a cox proportional-hazards regression mode. In multivariate analysis, presence of CSRN were significantly associated with PFS (hazard ratio [HR] 1.848; 95% CI 1.05–3.24; *p* = 0.032). Table 4 shows the risk factors associated with OS using a cox proportional-hazards regression mode. In multivariate analysis, albumin and presence of CSRN were significantly associated with OS (HR 2.401; 95% CI 1.073–5.370; *p* = 0.033 and HR 2.107; 95% CI 1.130–3.599; *p* = 0.018, respectively).

**Table 3:** Univariate and multivariate analyses of factors affecting PFS

		Univariate analysis		Multivariate analysis	
Variable		HR (95% CI)	***P*** value	HR (95% CI)	***P*** value
CSRN	**–**	**1.000**		**1.000**	
	**+**	**1.848 (1.054–3.242)**	**0.032**	**1.848 (1.054–3.242)**	**0.032**
Age	**< 70**	**1.000**			
	**≥ 70**	**1.812 (0.991–3.311)**	**0.054**		
Body weight (kg)	**< 60**	**1.000**			
	**≥ 60**	**1.089 (0.627–1.894)**	**0.761**		
Barcelona Clinic Liver Cancer stage	**B**	**1.000**			
	**C**	**1.498 (0.864–2.597)**	**0.150**		
Up to seven	**In**	**1.000**			
	**Out**	**1.243 (0.719–2.148)**	**0.436**		
Metastatic disease	**–**	**1.000**			
	**+**	**1.247 (0.703–2.214)**	**0.450**		
Previous therapy: Transcatheter treatment	**–**	**1.000**			
	**+**	**1.412 (0.761–2.623)**	**0.274**		
Refractory to Transcatheter treatment	**–**	**1.000**			
	**+**	**1.389 (0.501–2.123)**	**0.360**		
α-fetoprotein (ng/mL)	**< 400**	**1.000**			
	**≥ 400**	**1.100 (0.620–1.950)**	**0.745**		
Albumin	**≥ 3.2**	**1.000**			
	**< 3.2**	**2.130 (0.992–4.574)**	**0.052**

**Table 4:** Univariate and multivariate analyses of factors affecting overall survival

		Univariate analysis		Multivariate analysis	
Variable		HR (95% CI)	***P*** value	HR (95% CI)	***P*** value
CSRN	**–**	**1.000**		**1.000**	
	**+**	**2.203 (1.253–3.873)**	**0.006**	**2.017 (1.130–3.599)**	**0.018**
Age	**< 70**	**1.000**			
	**≥ 70**	**1.690 (0.926–3.085)**	**0.088**		
Body weight (kg)	**< 60**	**1.000**			
	**≥ 60**	**1.130 (0.652–1.960)**	**0.663**		
Barcelona Clinic Liver Cancer stage	**B**	**1.000**			
	**C**	**1.071 (0.616–1.861)**	**0.808**		
Up to seven	**In**	**1.000**			
	**Out**	**1.450 (0.838–2.510)**	**0.184**		
Metastatic disease	**–**	**1.000**			
	**+**	**1.042 (0.576–1.886)**	**0.891**		
Previous therapy: Transcatheter treatment	**–**	**1.000**			
	**+**	**1.203 (0.650–2.229)**	**0.556**		
Refractory to Transcatheter treatment	**–**				
	**+**	**1.311 (0.596–1.520)**	**0.742**		
α-fetoprotein (ng/mL)	**< 400**	**1.000**			
	**≥ 400**	**1.630 (0.920–2.885)**	**0.094**		
Albumin	**≥ 3.2**	**1.000**		**1.000**	
	**< 3.2**	**2.866 (1.309–6.276)**	**0.008**	**2.401 (1.073–5.370)**	**0.033**

## Discussion

To our knowledge, this is the first study to report the influence of NO levels after administration of lenvatinib regarding the clinical response and AEs for patients with unresectable HCC. A review clearly showed a significant reduction in NO levels after RFA for HCC [14]. Therefore, we expected a correlation between the change in NO levels and the patients’ responses to lenvatinib. In fact, a few cases showed positive correlations between increased NO levels and disease progression after the lenvatinib administration. Disease progression may be seen if HCC has higher production of serum NO levels than the restored NO production due to the lenvatinib therapy. However, there were no statistically significant correlations observed between the patients’ responses and the NO levels. The major difficulties in investigating the physiological role of NO is the direct measurement of NO due to its short lifetime and very low concentrations [17]. Therefore, most researchers refer to indirect qualitative measurements, such as the detection of NO-induced physiological AEs and employment of NOS inhibitors [18]. In this experiment, the Griess method based on the chemical diazotization reaction to detect NO, which is the indirect method based on two stable breakdown products, NO_3_^***−***^ and NO_2_^***−***^, can be easily detected by photometric means [19]. However, quantification by absorbance is affected by thiols and proteins. As a result, the indirect measurement may be slightly less rigorous than the direct measurement. In addition, we measured systemic NO in whole blood, including NO from tumor cells, hepatic sinusoidal endothelial cells, and vascular epithelial cells. The results of the present study may be different from those using real-time NO levels in tumor cells [20, 21].

On the other hand, the present study revealed that a significant reduction in the NO rate was associated with AEs after the lenvatinib therapy. Lenvatinib therapy improves HCC control by restoring the NO levels in tumor. However, excessively restored NO levels lead to the occurrence of AEs and shortened lengths of survival. Inhibition of angiogenic factor-mediated pathways, including VEGF, results in a subsequent reduction in NO production [22]. NO is an important vasodilator that maintains vascular tone by activating guanylate cyclase in vascular smooth muscle [23]. In hepatic microcirculation, the deficiency of endothelial NO release causes hemodynamic abnormalities and portal hypotension according to the progression of fibrosis in chronic liver disease [24]. As a result, a significant reduction in NO appears to promote the occurrence of AEs in anti-angiogenic therapy. In fact, our team revealed that lenvatinib aggravates portal hypertension using duplex Doppler ultrasonography [25].

We also expected a correlation between the change in NO levels and HBP. Previous studies have shown that patients who experienced hypertension after lenvatinib administration had significantly better outcomes than those who did not develop AEs [15]. A subsequent reduction in NO production lead to HBP. However, no correlation was observed between the occurrence of HBP and NO levels in the present study. Some patients in the present study had HBP. To provide treatment for the HBP may have masked the HBP caused by the lenvatinib therapy.

Thus, the present study revealed the correlation between the change rate of NO and SAEs in patients with HCC who underwent the lenvatinib therapy. However, as evidenced previously, the distinct roles of NO in patients treated with VEGF inhibitors remain unclear [26]. Previously, the relationship between NO levels and tumor progression has been linked to the presence of NOS enzymes in cells and/or serum. Therefore, NOS should be considered for patients with HCC who have received lenvatinib therapy. NOS is classified into three subgroups: neuronal NOS (nNOS), inducible NOS (iNOS), and endothelial NOS (eNOS). VEGF inhibitors mainly block the exposure of endothelial cells to VEGF, leading to the activation of eNOS, which is a signaling mechanism with prominent pathways in angiogenesis [27, 28]. eNOS is mainly expressed in liver sinusoidal endothelial cells and vascular epithelial cells, including the hepatic artery, central veins, and portal vein. The most common cause of portal hypertension is an increase in intrahepatic vascular resistance through the production of eNOS-derived NO [29]. The excessive reduction of eNOS by lenvatinib leads to AEs. Furthermore, it will be necessary to verify the influence of iNOS on the HCC response in future studies. Tumor cells are the primary sites for excess NO production, and the amount of NO produced by iNOS, which is higher than that produced by nNOS or eNOS, contributes to tumor cell-related angiogenesis, malignant transformation, invasion, and metastasis [30, 31]. The overproduction of NO in malignant tissues by iNOS inhibits the immune defense mechanism and increases tumor blood, correlating with carcinogenesis and playing a role in tumor progression in HCC [32, 33]. Therefore, it is necessary to consider not only NO but also NOS in patients with HCC.

There are a few limitations to this study. First, it was a retrospective study. Second, preserved blood serum samples could not be obtained on a scheduled day from all the patients receiving lenvatinib. Furthermore, we need to evaluate the most appropriate time after administration of lenvatinib in which AEs occur. Third, NO was indirectly assessed using nitrate NO_3_^***−***^ and nitrite NO_2_^***−***^. Evaluation of NO by direct methods will be needed for future analysis. We only evaluated the correlation between the change rate of the NO levels and the clinical responses and AEs in the present study. Furthermore, this study did not exclude the effect on NO concentrations from any treatment given before the lenvatinib administration. There is insufficient evidence in the literature on the mechanism of how NO affects the therapeutic response and AEs. Fourth, patients with a total bilirubin of 2.0 mg/dL or higher were excluded because of the effect on absorbance measurements.

## Conclusion

Decreased NO levels were associated with the occurrence of AEs and worse prognosis after the lenvatinib administration. Change rate in serum NO levels can be used as predictive markers in patients receiving lenvatinib therapy for HCC.

## Supplementary Information

**Additional file 1.** The measurement of NO Production of NO by NOS and its metabolism to nitrate and nitrite. Nitrate was converted to nitrite by nitrate reductase, and NO was measured using the Griess reagent. The microtiter plate after the reaction is shown. (PPTX 2781 kb)

**Additional file 2.** Pretreatment NO levels

**Additional file 3.** ROC. The occurrence of SAE using lenvatinib by ROC curves for patients with HCC. AUC, area under the curve. (PPTX 48 kb)

## Data Availability

The data that support the findings of this study are available from Haruki Uojima but restrictions apply to the availability of these data, which were used under license for the current study, and so are not publicly available. Data are however available from the authors upon reasonable request and with permission of Haruki Uojima.

## Abbreviations

NO: Nitric oxide
AE: Adverse event
HCC: Hepatocellular carcinoma
NO_3_^−^: Nitrate _3_^−^
NO_2_^−^: Nitrite _2_^−^
CSRN: Clinically significant reduction of nitric oxide
PFS: Progression-free survival
OS: Overall survival
NOS: Nitric oxide synthase
nNOS: Neuronal nitric oxide synthase
iNOS: Inducible nitric oxide synthase
eNOS: endothelial nitric oxide synthase
VEGF: Vascular endothelial growth factor
mRECIST: modified Response Evaluation Criteria in Solid Tumor
CI: Confidence interval
RFA: Radiofrequency ablation
BCLC: Barcelona clinic liver cancer
PD: Progressive disease
HBP: High blood pressure
ROC: Receiver-operating characteristic
ALBI: Albumin-bilirubin

## References

[1] Abu-Amara M, Yang SY, Seifalian A, Davidson B, Fuller B (2012). The nitric oxide pathway – evidence and mechanisms for protection against liver ischaemia reperfusion injury. Liver Int.

[2] Iwakiri Y, Kim MY (2015). Nitric oxide in liver diseases. Pharmacol Sci.

[3] Hurshman AR, Marletta MA (1995). Nitric oxide complexes of inducible nitric oxide synthase: spectral characterization and effect on catalytic activity. Biochemistry..

[4] Apte RS, Chen DS, Ferrara N (2019). VEGF in signaling and disease: beyond discovery and development. Cell..

[5] Carmeliet P (2005). VEGF as a key mediator of angiogenesis in cancer. Oncology..

[6] Ziche M, Morbidelli L (2009). Molecular regulation of tumour angiogenesis by nitric oxide. Eur Cytokine Netw.

[7] Kudo M (2018). Lenvatinib may drastically change the treatment landscape of hepatocellular carcinoma. Liver Cancer.

[8] Kudo M, Ueshima K, Chan S, Minami T, Chishina H, Aoki T (2019). Lenvatinib as an initial treatment in patients with intermediate-stage hepatocellular carcinoma beyond up-to-seven criteria and child-Pugh a liver function: a proof-of-concept study. Cancers (Basel).

[9] Kudo M (2019). A new treatment option for intermediate-stage hepatocellular carcinoma with high tumor burden: initial lenvatinib therapy with subsequent selective TACE. Liver Cancer.

[10] Chen CH, Wu SH, Tseng YM, Hou MF, Tsai LY, Tsai SM (2018). Distinct role of endothelial nitric oxide synthase gene polymorphisms from menopausal status in the patients with sporadic breast cancer in Taiwan. Nitric Oxide.

[11] Lencioni R, Llovet JM (2010). Modified RECIST (mRECIST) assessment for hepatocellular carcinoma. Semin Liver Dis.

[12] Kudo M, Finn RS, Qin S, Han KH, Ikeda K, Piscaglia F (2018). Lenvatinib versus sorafenib in first-line treatment of patients with unresectable hepatocellular carcinoma: a randomised phase 3 non-inferiority trial. Lancet..

[13] Moety HAAE, Moety AAE, Sayed PE (2013). Evaluation of serum nitric oxide before and after local radiofrequency thermal ablation for hepatocellular carcinoma. Alexandria J Med.

[14] Robbins RA, Grisham MB (1997). Nitric oxide. Int J Biochem Cell Biol.

[15] Azuma S, Uojima H, Chuma M, Shao X, Hidaka H, Nakazawa T (2020). Influence of NOS3 rs2070744 genotypes on hepatocellular carcinoma patients treated with lenvatinib. Sci Rep.

[16] Finn RS, Kudo M, Cheng AL, Wyrwicz L, Ngan RKC, Blanc JF (2021). Pharmacodynamic biomarkers predictive of survival benefit with Lenvatinib in Unresectable hepatocellular carcinoma: from the phase III REFLECT study. Clin Cancer Res.

[17] Zhou L, Wang Y, Tian DA, Yang J, Yang YZ (2012). Decreased levels of nitric oxide production and nitric oxide synthase-2 expression are associated with the development and metastasis of hepatocellular carcinoma. Mol Med Rep.

[18] Taylor BS, Alarcon LH, Billiar TR (1998). Inducible nitric oxide synthase in the liver: regulation and function. Biochemistry (Mosc).

[19] Zhang XG, Jin L, Tian Z, Wang Y, Liu JF, Chen Y (2019). Nitric oxide inhibits autophagy and promotes apoptosis in hepatocellular carcinoma. Cancer Sci.

[20] Romitelli F, Santini SA, Chierici E, Pitocco D, Tavazzi B, Amorini AM (2007). Comparison of nitrite/nitrate concentration in human plasma and serum samples measured by the enzymatic batch Griess assay, ion-pairing HPLC and ion-trap GC-MS: the importance of a correct removal of proteins in the Griess assay. J Chromatogr B Analyt Technol Biomed Life Sci.

[21] Tsikas D, Gutzki FM, Rossa S, Bauer H, Neumann C, Dockendorff K (1997). Measurement of nitrite and nitrate in biological fluids by gas chromatography–mass spectrometry and by the Griess assay: problems with the Griess assay–solutions by gas chromatography–mass spectrometry. Anal Biochem.

[22] Pârvu AE, Negrean V, Pleşca-Manea L, Cosma A, Draghici A, Uifalean A (2005). Nitric oxide in patients with chronic liver diseases. Rom J Gastroenterol.

[23] Atucha NM, Nadal FJA, Iyú D, Alcaraz A, Rodriguez-Barbero A, Ortiz MC (2005). Role of vascular nitric oxide in experimental liver cirrhosis. Curr Vasc Pharmacol.

[24] Abrams GA, Trauner M, Nathanson MH (1995). Nitric oxide and liver disease. Gastroenterologist..

[25] Ohya K, Kawaoka T, Namba M, Uchikawa S, Kodama K, Morio K (2019). Early changes in ammonia levels and liver function in patients with advanced hepatocellular carcinoma treated by lenvatinib therapy. Sci Rep.

[26] Hidaka H, Uojima H, Nakazawa T, Sho X, Hara Y, Iwasaki S (2020). Portal hemodynamic effects of lenvatinib in patients with advanced hepatocellular carcinoma: a prospective cohort study. Hepatol Res.

[27] Farzaneh-Far R, Moore K (2001). Nitric oxide and the liver. Liver..

[28] Hu LS, George J, Wang JH (2013). Current concepts on the role of nitric oxide in portal hypertension. World J Gastroenterol.

[29] Rao VLR (2002). Nitric oxide in hepatic encephalopathy and hyperammonemia. Neurochem Int.

[30] Filik L (2011). Nitric oxide levels in cirrhotic patients with hepatic encephalopathy. J Clin Gastroenterol.

[31] Tache DE, Stanciulescu CE, Banita IM, Purcaru SO, Andrei AM, Comanescu V (2014). Inducible nitric oxide synthase expression (iNOS) in chronic viral hepatitis and its correlation with liver fibrosis. Romanian J Morphol Embryol.

[32] Anavi S, Tirosh O (2020). iNOS as a metabolic enzyme under stress conditions. Free Radic Biol Med.

[33] La Mura V, Pasarín M, Rodriguez-Vilarrupla A, García-Pagán JC, Bosch J, Abraldes JG (2014). Liver sinusoidal endothelial dysfunction after LPS administration: a role for inducible-nitric oxide synthase. J Hepatol.

