# Supplementary figures and images for: The change rate in serum nitric oxide may affect lenvatinib therapy in hepatocellular carcinoma
Source: BMC Cancer. 2022 Aug 23;22:912. doi: 10.1186/s12885-022-10002-x (PMC9396897; doi:10.1186/s12885-022-10002-x)

## Slide 1
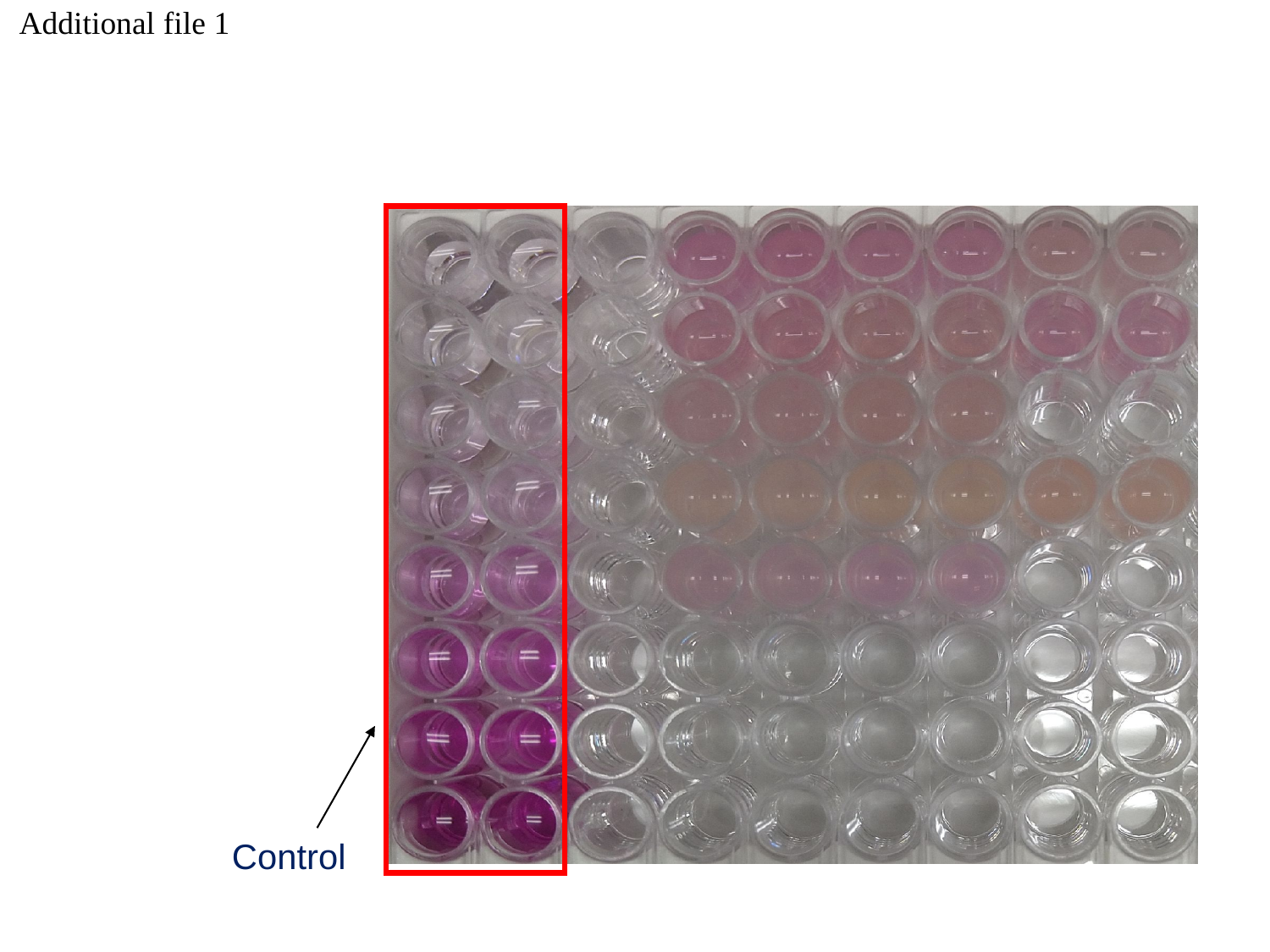

Additional file 1
Control

Supplement: Supplementary file 1 — Additional file 1. The measurement of NO Production of NO by NOS and its metabolism to nitrate and nitrite. Nitrate was converted to nitrite by nitrate reductase, and NO was measured using the Griess reagent. The microtiter plate after the reaction is shown. (PPTX 2781 kb) [file 12885_2022_10002_MOESM1_ESM.pptx]
