# Supplementary material for: The change rate in serum nitric oxide may affect lenvatinib therapy in hepatocellular carcinoma
Source: BMC Cancer. 2022 Aug 23;22:912. doi: 10.1186/s12885-022-10002-x (PMC9396897; doi:10.1186/s12885-022-10002-x)
Supplement: Supplementary file 2 — Additional file 2. Pretreatment NO levels [file 12885_2022_10002_MOESM2_ESM.docx]

**Additional file 2. Pretreatment NO levels**

| Variable | NO levels (nmol/mL) | | *P* value |
| --- | --- | --- | --- |
| Age > 70/< 70 | 43.9 ± 39.1 | 53.7 ± 40.7 | 0.309 |
| Gender: Male/Female | 49.8 ± 39.7 | 47.2 ± 41.7 | 0.811 |
| Etiology: Virus/Non-virus | 53.7 ± 40.5 | 43.9 ± 39.1 | 0.307 |
| Child-Pugh class: A/B | 33.2 ± 21.2 | 41.2 ± 20.2 | 0.332 |
| Barcelona Clinic Liver Cancer stage: B/C | 48.3 ± 40.5 | 50.7 ± 40.1 | 0.806 |
| Macroscopic portal vein invasion: Yes/No | 52.6 ± 42.2 | 47.4 ± 38.9 | 0.611 |
| Up to 7: In/Out | 46.6 ± 39.6 | 52.0 ± 40.6 | 0.569 |
| Extrahepatic spread: Yes/No | 59.6 ± 49.2 | 44.4 ± 34.3 | 0.141 |
| Hemoglobin: < 10/≧10 | 56.4 ± 41.6 | 47.9 ± 39.8 | 0.518 |
| Prothrombin time: < 70≧70 | 36.4 ± 20.6 | 51.6 ± 42.2 | 0.248 |
| Serum albumin: < 3.5/≧3.5 | 54.1 ± 35.1 | 46.7 ± 42.3 | 0.467 |
| Alanine aminotransferase: < 30/≧30 | 49.2 ± 43.4 | 49.3 ± 36.1 | 0.991 |
| Total bilirubin: < 1/≧1 | 44.4 ± 33.1 | 59.0 ± 50.4 | 0.152 |
| α-fetoprotein: < 400/≧400 | 48.5 ± 40.6 | 50.5 ± 39.2 | 0.847 |
