# Supplementary material for: The change rate in serum nitric oxide may affect lenvatinib therapy in hepatocellular carcinoma
Source: BMC Cancer. 2022 Aug 23;22:912. doi: 10.1186/s12885-022-10002-x (PMC9396897; doi:10.1186/s12885-022-10002-x)
Supplement: Supplementary file 3 — Additional file 3. ROC. The occurrence of SAE using lenvatinib by ROC curves for patients with HCC. AUC, area under the curve. (PPTX 48 kb) [file 12885_2022_10002_MOESM3_ESM.pptx]

## Slide 1
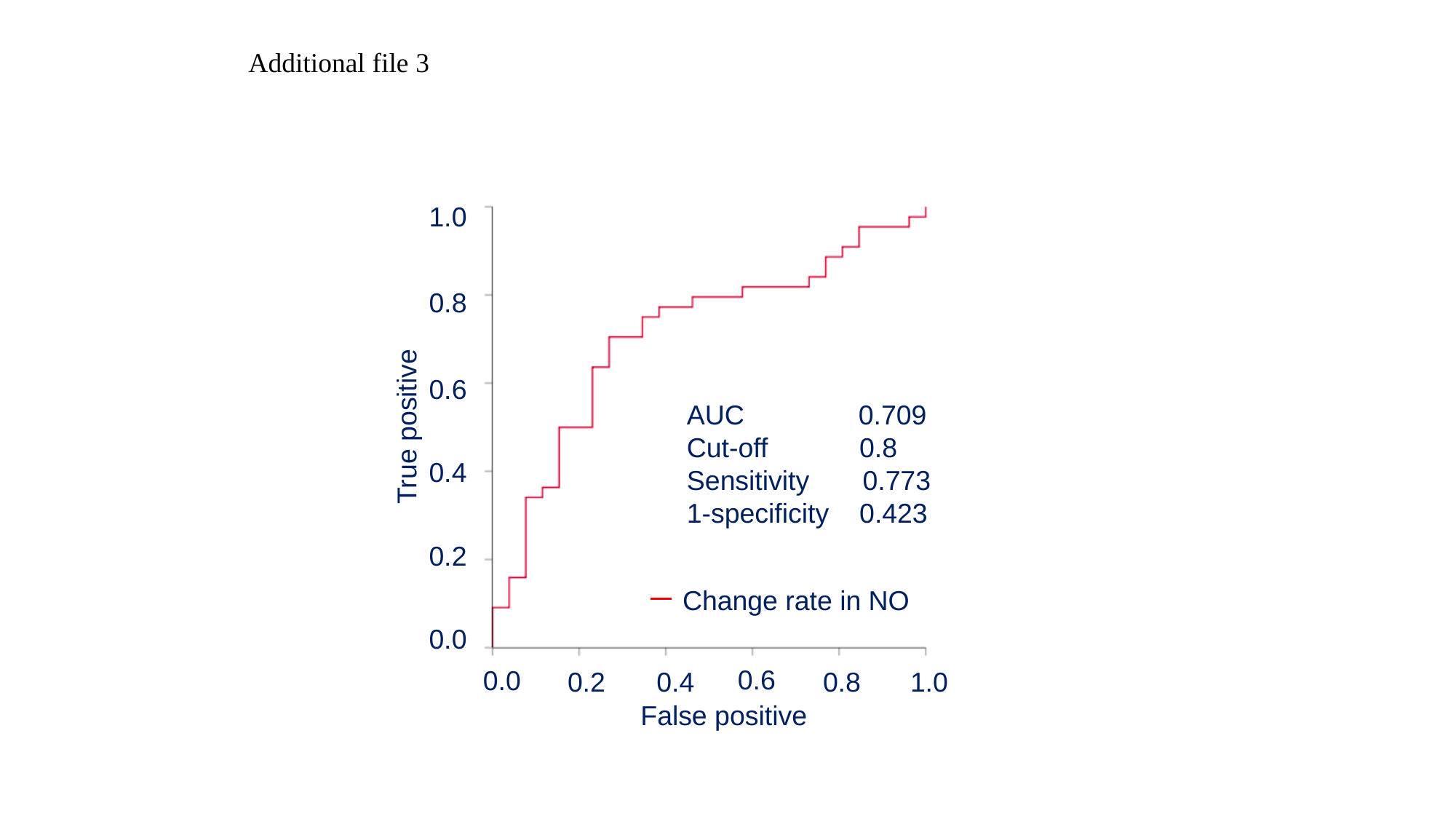

Additional file 3
1.0
0.8
0.6
AUC 0.709
Cut-off 0.8
Sensitivity 0.773
1-specificity 0.423
True positive
0.4
0.2
Change rate in NO
0.0
0.6
0.0
0.2
0.4
0.8
1.0
False positive
